# Supplementary material for: Molecular Identification and Prevalence of the Mite Carpoglyphus lactis (Acarina: Carpoglyphidae) in Apis mellifera in the Republic of Korea
Source: Insects. 2024 Apr 14;15(4):271. doi: 10.3390/insects15040271 (PMC11050302; doi:10.3390/insects15040271)
Supplement: Supplementary file 1 [file insects-15-00271-s001.zip › insects-2936428-supplementary.pdf]

Alignment: C:\BioEdit\Temp\~out.tmp

```
               ....|....| ....|....| ....|....| ....|....| ....|....| ....|....| ....|....| ....|....| ....|....| ....|....|
                      10         20         30         40         50         60         70         80         90        100
MN073839.1-Ch  CTAGTCTTAG ATCCGTTATT CGTTTTGAGC TTACTGTTCC TGGGGACTAT CTGATAGACT ACGATTATTA CAACTCAGTA GTTACAGCCC ATGCTTTTGT
NC048990.1-Ch  .......... .......... .......... .......... .......... .......... .......... .......... .......... ..........
KY922482.1-UK  .......... .......... .......... .......... ......T... .......... .......... .......... .......... ..........
GN-KOR1        .......... .......... .......... .......... ......T... .......... .......... .......... .......... ..........
JN-KOR2        .......... .......... .......... .......... ......T... .......... .......... .........G .......... ..........
GN-KOR3        .......... .......... .......... .......... ......T... .......... .......... .......... .......... ..........
GB-KOR1        .......... .......... .......... .......... ......T... .......... .......... .......... .......... ..........
JN-KOR4        .......... .......... .......... .......... ......T... .......... .......... .......... .......... ..........
JN-KOR1        .......... .......... .......... .......... .......... .......... .......... .......... .......... ..........
GG-KOR1        .......... .......... .......... .......... ......T... .......... .......... .......... .......... ..........
CN-KOR1        .......... .......... .......... .......... ......T... .......... .......... .........G .......... ..........
CB-KOR1        .......... .......... .......... .......... ......T... .....G.... .......... ......C... .......... ..........
CB-KOR2        .......... .......... .......... .......... ......T... .......... .......... .......... .......... ..........
JN-KOR3        .......... .......... .......... .......... .......... .......... .......... .........G .......... ..........
GB-KOR2        .......... .......... .......... .......... ......T... .......... .......... .......... .......... ..........
GN-KOR2        .......... .......... .......... .......... .......... .......... .......... .......... .......... ..........
JB-KOR2        .......... .......... .......... .......... ......T... .......... .......... .......... .......... ..........
JB-KOR1        .......... .......... .......... .......... ......T... .......... .......... .......... .......... ..........
GW-KOR1        .......... .......... .......... .......... ......T... .......... .......... .......... .......... ..........
CN-KOR2        .......... .......... .......... .......... ......T... .......... .......... .......... .......... ..........
CN-KOR3        .......... .......... .......... .......... ......T... .......... .......... .......... .......... ..........
JB-KOR3        .......... .......... .......... .......... ......T... .......... .......... .......... .......... ..........

               ....|....| ....|....| ....|....| ....|....| ....|....| ....|....| ....|....| ....|....| ....|....| ....|....|
                     110        120        130        140        150        160        170        180        190        200
MN073839.1-Ch  TATAATTTTT TTCATAGTAA TACCTATTAT AATAGGAGGG TTTGGGAACC TTCTTGTTCC AGTTATAATT GGGGCTATAG ATATAGCTTA CCCTCGTCTT
NC048990.1-Ch  .......... .......... .......... .......... .......... .......... .......... .......... .......... ..........
KY922482.1-UK  .......... .......... .......... .......... .......... .......... .......... .......... .......... ..........
GN-KOR1        .......... .......... .......... .......... .......... .......... .......... .......... .......... ..........
JN-KOR2        .......... .......... .......... .......... .......... .......... .......... .......... .......... ..........
GN-KOR3        .......... .......... .......... .......... .......... .......... .......... .......... .......... ..........
GB-KOR1        .......... .......... .......... .......... .......... .......... .......... .......... .......... ..........
JN-KOR4        .......... .......... .......... .......... .......... .......... .......... .......... .......... ..........
JN-KOR1        .......... .......... .......... .......... .......... .......... .......... .......... .......... ..........
GG-KOR1        .......... .......... .......... .......... .......... .......... .......... .......... .......... ..........
CN-KOR1        .......... .......... .......... .......... .......... .......... .......... .......... .......... ..........
CB-KOR1        .......... .......... .......... .......... .......... .......... .......... .......... .......... ..........
CB-KOR2        .......... .......... .......... .......... .......... .......... .......... .......... .......... ..........
JN-KOR3        .......... .......... .......... .......... .......... .......... .......... .......... .......... ..........
GB-KOR2        .......... .......... .......... .......... .......... .......... .......... .......... .......... ..........
GN-KOR2        .......... .......... .......... .......... .......... .......... .......... .......... .......... ..........
JB-KOR2        .......... .......... .......... .......... .......... .......... .......... .......... .......... ..........
JB-KOR1        .......... .......... .......... .......... .......... .......... .......... .......... .......... ..........
GW-KOR1        .......... .......... .......... .......... .......... .......... .......... .......... .......... ..........
CN-KOR2        .......... .......... .......... .......... .......... .......... .......... .......... .......... ..........
CN-KOR3        .......... .......... .......... .......... .......... .......... .......... .......... .......... ..........
JB-KOR3        .......... .......... .......... .......... .......... .......... .......... .......... .......... ..........

               ....|....| ....|....| ....|....| ....|....| ....|....| ....|....| ....|....| ....|....| ....|....| ....|....|
                     210        220        230        240        250        260        270        280        290        300
MN073839.1-Ch  AATAATATAA GGTTTTGACT TTTGCCTCCT TCTTTATCTT TGCTTATCAG ATCTGCTGTA GTGGGTTCTG GTGTAGGTAC AGGATGAACT GTTTATCCTC
NC048990.1-Ch  .......... .......... .......... .......... .......... .......... .......... .......... .......... ..........
KY922482.1-UK  .......... .......... .......... .......... .......... .......... .......... ....G..... .......... ..........
GN-KOR1        .......... .......... .......... .......... .......... .......... .......... .......... .......... ..........
JN-KOR2        .......... .......... .......... .......... .......... .......... .......... .......... .......... ..........
GN-KOR3        .......... .......... .......... .......... .......... .......... .......... .......... .......... ..........
GB-KOR1        .......... .......... .......... .......... .......... .......... .......... .......... .......... ..........
JN-KOR4        .......... .......... .......... .......... .......... .......... .......... .......... .......... ..........
JN-KOR1        .......... .......... .......... .......... .......... .......... .......... ....G..... .......... ..........
GG-KOR1        .......... .......... .......... .......... .......... .......... .......... .......... .......... ..........
CN-KOR1        .......... .......... .......... .......... .......... .......... .......... .......... .......... ..........
CB-KOR1        .......... .......... .......... .......... .......... .......... .......... .......... .......... ..........
CB-KOR2        .......... .......... .......... .......... .......... .......... .......... .......... .......... ..........
JN-KOR3        .......... .......... .......... .......... .......... .......... .......... .......... .......... ..........
GB-KOR2        .......... .......... .......... .......... .......... .......... .......... .......... .......... ..........
GN-KOR2        .......... .......... .......... .......... .......... .......... .......... .......... .......... ..........
JB-KOR2        .......... .......... .......... .......... .......... .......... .......... .......... .......... ..........
JB-KOR1        .......... .......... .......... .......... .......... .......... .......... .......... .......... ..........
GW-KOR1        .......... .......... .......... .......... .......... .......... .......... .......... .......... ..........
CN-KOR2        .......... .......... .......... .......... .......... .......... .......... .......... .......... ..........
CN-KOR3        .......... .......... .......... .......... .......... .......... .......... .......... .......... ..........
JB-KOR3        .......... .......... .......... .......... .......... .......... .......... .......... .......... ..........

               ....|....| ....|....| ....|....| ....|....| ....|....| ....|....| ....|....| ....|....| ....|....| ....|....|
                     310        320        330        340        350        360        370        380        390        400
MN073839.1-Ch  CTCTTTCTAA TGGAATTTTC CATTCAGGCC CTGCTGTAGA TTTTGGTATC CTTAGTTTAC ATGTAGCTGG GGTCTCTTCA ATTTTAGGTG CTATTAATTT
NC048990.1-Ch  .......... .......... .......... .......... .......... .......... .......... .......... .......... ..........
KY922482.1-UK  .......... .......... .......... .......... .........T .......... .......... .......... .......... ..........
GN-KOR1        .......... .......... .......... .......... .......... .......... .......... .......... .......... ..........
JN-KOR2        .......... .......... .......... .......... .......... .......... .......... .......... .......... ..........
GN-KOR3        .......... .......... .......... .......... .......... .......... .......... .......... .......... ..........
GB-KOR1        .......... .......... .......... .......... .......... .......... .......... .......... .......... ..........
JN-KOR4        .......... .......... .......... .......... .......... .......... .......... .......... .......... ..........
JN-KOR1        .......... .......... .......... .......... .......... .......... .......... .......... .......... ..........
GG-KOR1        .......... .......... .......... .......... .........T .......... .......... .......... .......... ..........
CN-KOR1        .......... .......... .......... .......... .......... .......... .......... .......... ..G....... ..........
CB-KOR1        .......... .......... .......... .......... .......... .......... .......... .......... .......... ..........
CB-KOR2        .......... .......... .......... .......... .......... .......... .......... .......... .......... ..........
JN-KOR3        .......... .......... .......... .......... .......... .......... .......... .......... .......... ..........
GB-KOR2        .......... .......... .......... .......... .......... .......... .......... .......... .......... ..........
GN-KOR2        .......... .......... .......... .......... .......... .......... .......... .......... .......... ..........
JB-KOR2        .......... .......... .......... .......... .......... .......... .......... .......... .......... ..........
JB-KOR1        .......... .......... .......... .......... .......... .......... .......... .......... .......... ..........
GW-KOR1        .......... .......... .......... .......... .......... .......... .......... .......... .......... ..........
CN-KOR2        .......... .......... .......... .......... .......... .......... .......... .......... .......... ..........
CN-KOR3        .......... .......... .......... .......... .......... .......... .......... .......... .......... ..........
JB-KOR3        .......... .......... .......... .......... .......... .......... .......... .......... .......... ..........
```

```
               ....|....| ....|....| ....|....| ....|....| ....|....| ....|....| ....|....| ....|....| ....|....| ....|....|
                     410        420        430        440        450        460        470        480        490        500
MN073839.1-Ch  TATTGTCACT ATTTTTAACA TAAAAACTTT AGGCATAAGA TGGTCTAATG TTCCTTTATT TGTTTGATCT GTTTTGATTA CTTCTTTCCT CTTAGCTTTT
NC048990.1-Ch  .......... .......... .......... .......... .......... .......... .......... .......... .......... ..........
KY922482.1-UK  .......... .......... .......... ...T...... ..A....... .......... .......... .......... .......... ..........
GN-KOR1        .......... .......T.. .......... ..T....... .......... .......... .......... .......... .......... ..........
JN-KOR2        .......... ....C..... .......... ..T....... .A........ .......... .......... .......... .......... ..........
GN-KOR3        .......... ....C..... .......... ..T....... .A........ .......... .......... .......... .......... ..........
GB-KOR1        .......... ....C..... .......... ..T....... .A........ .......... .......... .......... .......... ..........
JN-KOR4        .......... ....C..... .......... ..T....... .A........ .......... .......... .......... .......... ..........
JN-KOR1        .......... ....C..... .......... ..T....... .A........ .......... .......... .......... .......... ..........
GG-KOR1        .......... ....C..... .......... ..T....... .A........ .......... .......... .......... .......... ..........
CN-KOR1        .......... .......... .......... ..T....... .A........ .......... .......... .......... .......... ..........
CB-KOR1        .......... ....C..... .......... ..T....... .A........ .......... .......... .......... .......... ..........
CB-KOR2        .......... ....C..... .......... ..T....... .A........ .......... .......... .......... .......... ..........
JN-KOR3        .......... ....C..... .......... ..T....... .A........ .......... .......... .......... .......... ..........
GB-KOR2        .......... ....C..... .......... ..T....... .A........ .......... .......... .......... .......... ..........
GN-KOR2        .......... ....C..... .......... ..T....... .......... .......... .......... .......... .......... ..........
JB-KOR2        .......... ....C..... .......... ..T....... .A........ .......... .......... .......... .......... ..........
JB-KOR1        .......... ....C..... .......... ..T....... .A........ .......... .......... .......... .......... ..........
GW-KOR1        .......... ....C..... .......... ..T....... .A........ .......... .......... .......... .......... ..........
CN-KOR2        .......... ....C..... .......... ..T....... .......... .......... .......... .......... .......... ..........
CN-KOR3        .......... ....C..... .......... ..T....... .A........ .......... .......... .......... .......... ..........
JB-KOR3        .......... ....C..... .......... ..T....... .A........ .......... .......... .......... .......... ..........

               ....|....| ....|....| ....|....| ....|....| ....|....| ....|....| ....|....| ....|....| ....|....| ....|....|
                     510        520        530        540        550        560        570        580        590        600
MN073839.1-Ch  TCTTTACCTG TATTAGCTGC TGCTCTTACT ATACTATTAA CAGATCGTAA TTTTAACACT ACTTTTTTTG ACCCTGTAGG AGGAGGTGAC CCTATCCTCT
NC048990.1-Ch  .......... .......... .......... .......... .......... .......... .......... .......... .......... ..........
KY922482.1-UK  .......... .......... .......... .......... .......... .......... .......... .......... .......... ........T.
GN-KOR1        .......... .......... .......... .......... .......... .......... .......... .......... .......... ..........
JN-KOR2        .......... .......... .......... .......... .......... .......... .......... .......... .......... ..........
GN-KOR3        .......... .......... .......... .......... .......... .......... .......... .......... .......... ..........
GB-KOR1        .......... .......... .......... .......... .......... .......... .......... .......... .......... ..........
JN-KOR4        .......... .......... .......... .......... .......... .......... .......... .......... .......... ..........
JN-KOR1        .......... .......... .......... .......... .......... .......... .......... .......... .......... ..........
GG-KOR1        .......... .......... .......... .......... .......... .......... .......... .......... .......... ..........
CN-KOR1        .......... .......... .......... .......... .......... .......... .......... .......... .......... ..........
CB-KOR1        .......... .......... .......... .......... .......... .......... .......... .......... .......... ..........
CB-KOR2        .......... .......... .......... .......... .......... .......... .......... .......... .......... ..........
JN-KOR3        .......... .......... .......... .......... .......... .......... .......... .......... .......... ..........
GB-KOR2        .......... .......... .......... .......... .......... .......... .......... .......... .......... ..........
GN-KOR2        .......... .......... .......... .......... .......... .......... .......... .......... .......... ..........
JB-KOR2        .......... .......... .......... .......... .......... .......... .......... .......... .......... ..........
JB-KOR1        .......... .......... .......... .......... .......... .......... .......... .......... .......... ..........
GW-KOR1        .......... .......... .......... .......... .......... .......... .......... .......... .......... ..........
CN-KOR2        .......... .......... .......... .......... .......... .......... .......... .......... .......... ..........
CN-KOR3        .......... .......... .......... .......... .......... .......... .......... .......... .......... ........T.
JB-KOR3        .......... .......... .......... .......... .......... .......... .......... .......... .......... ..........

               ....|....| ....|....| ....|....| ....|....| ....|....| ....|....| ....|....| ....|....| ....|....| ....|....|
                     610        620        630        640        650        660        670        680        690        700
MN073839.1-Ch  ACCAACATCT TTTCTGATTC TTTGGCCACC CAGAGGTTTA TATTCTTATT TTACCAGGGT TTGGTATTAT TTCTCATACA GTTATAACTT ACAGAAATAA
NC048990.1-Ch  .......... .......... .......... .......... .......... .......... .......... .......... .......... ..........
KY922482.1-UK  .T........ .......... .......... .......... .......... .......... .......... .......... .......... ..........
GN-KOR1        .......... .......... .......... .......... ...C...... .......... .......... .......... .......... ..........
JN-KOR2        .......... .......... .......... .......... .......... .......... .......... .......... .......... ..........
GN-KOR3        .......... .......... .......... .......... .......... .......... .......... .......... .....G.... ..........
GB-KOR1        .......... .......... .......... .......... .......... .......... .......... .......... .....G.... ..........
JN-KOR4        .......... .......... .......... .......... .......... .......... .......... .......... .....G.... ..........
JN-KOR1        .......... .......... .......... .......... .......... .......... .......... .......... .......... ..........
GG-KOR1        .......... .......... .......... .......... .......... .......... .......... .......... .....G.... ..........
CN-KOR1        .......... .......... .......... .......... .......... .......... .......... .......... .....G.... ..........
CB-KOR1        .......... .......... .......... .......... .......... .......... .......... .......... .......... ..........
CB-KOR2        .......... .......... .......... .......... .......... .......... .......... .......... .......... ..........
JN-KOR3        .......... .......... .......... .......... .......... .......... .......... .......... .......... ..........
GB-KOR2        .......... .......... .......... .......... .......... .......... .......... .......... .....G.... ..........
GN-KOR2        .......... .......... .......... .......... .......... .......... .......... .......... .......... ..........
JB-KOR2        .......... .......... .......... .......... .......... .......... .......... .......... .....G.... ..........
JB-KOR1        .......... .......... .......... .......... .......... .......... .......... .......... .....G.... ..........
GW-KOR1        .......... .......... .......... .......... .......... .......... .......... .......... .....G.... ..........
CN-KOR2        .......... .......... .......... .......... .......... .......... .......... .......... .......... ..........
CN-KOR3        .T........ .......... .......... .......... .......... .......... .......... .......... .......... ..........
JB-KOR3        .......... .......... .......... .......... .......... .......... .......... .......... .......... ..........

               ....|....| ....|....| ....|....| ....|....| ....|....| ....|....| ....|....| ....|....| ....|....| ....|....|
                     710        720        730        740        750        760        770        780        790        800
MN073839.1-Ch  AAGGGAACCT TTTGGAGGAT TGGGTATAAT TTATGCTATA GTTTCTATTG GGACCTTAGG TTTTATTGTG TGGGCTCACC ACATATTTAC TGTAGGCTTA
NC048990.1-Ch  .......... .......... .......... .......... .......... .......... .......... .......... .......... ..........
KY922482.1-UK  .......... .......... .......... .......... .......... .......... .......... .......... .......... ..........
GN-KOR1        .......... .......... .......... .......... .......... .......... .......... .......... .......... ..........
JN-KOR2        .......... .......... .......... .......... .......... .......... .......... .......... .......... ..........
GN-KOR3        .......... .......... .......... .......... .......... .......... .......... .......... .......... ..........
GB-KOR1        .......... .......... .......... .......... .......... .......... .......... .......... .......... ..........
JN-KOR4        .......... .......... .......... .......... .......... .......... .......... .......... .......... ..........
JN-KOR1        .......... .......... .......... .......... .......... .......... .......... .......... .......... ..........
GG-KOR1        .......... .......... .......... .......... .......... .......... .......... .......... .......... ..........
CN-KOR1        .......... .......... .......... .......... .......... .......... .......... .......... .......... ..........
CB-KOR1        .......... .......... .......... .......... .......... .......... .......... .......... .......... ..........
CB-KOR2        .......... .......... .......... .......... .......... .......... .......... .......... .......... ..........
JN-KOR3        .......... .......... .......... .......... .......... .......... .......... .......... .......... ..........
GB-KOR2        .......... .......... .......... .......... .......... .......... .......... .......... .......... ..........
GN-KOR2        .......... .......... .......... .......... .......... .......... .......... .......... .......... ..........
JB-KOR2        .......... .......... .......... .......... .......... .......... .......... .......... .......... ..........
JB-KOR1        .......... .......... .......... .......... .......... .......... .......... .......... .......... ..........
GW-KOR1        .......... .......... .......... .......... .......... .......... .......... .......... .......... ..........
CN-KOR2        .......... .......... .......... .......... .......... .......... .......... .......... .......... ..........
CN-KOR3        .......... .......... .......... .......... .......... .......... .......... .......... .......... ..........
JB-KOR3        .......... .......... .......... .......... .......... .......... .......... .......... .......... ..........
```

```
              ....|....| ....|....| ....|....| ....|....| ....|....| ....|....| ....|....| ....|....| ....|....| ....|....|
                    810        820        830        840        850        860        870        880        890        900
MN073839.1-Ch  GATGTTGACA CTCGAGCCTA TTTTACTGCT GCTACAATGA TTATTGCTGT TCCTACTGGG GTTAAAGTAT TTAGTTGGCT AGCTACTATA ATAGGAGGCT
NC048990.1-Ch  .......... .......... .......... .......... .......... .......... .......... .......... .......... ..........
KY922482.1-UK  .......... .......... .......... .......... .......... .......... .....G.... .......... .......... ..........
GN-KOR1        .......... .......... .......... .......... .......... .......... .......... .......... .......... ..........
JN-KOR2        .......... .......... .......... .......... .......... .......... .......... .......... .......... ..........
GN-KOR3        .......... .......... .......... .......... .......... .......... .......... .......... .......... ..........
GB-KOR1        .......... .......... .......... .......... .......... .......... .......... .......... .......... ..........
JN-KOR4        .......... .......... .......... .......... .......... .......... .......... .......... .......... ..........
JN-KOR1        .......... .......... .......... .......... .......... .......... .......... .......... .......... ..........
GG-KOR1        .......... .......... .......... .......... .......... .......... .....G.... .......... .......... ..........
CN-KOR1        .......... .......... .......... .......... .......... .......... .......... .......... .......... ..........
CB-KOR1        .......... .......... .......... .......... .......... .......... .......... .......... .......... ..........
CB-KOR2        .......... .......... .......... .......... .......... .......... .......... .......... .......... ..........
JN-KOR3        .......... .......... .......... .......... .......... .......... .......... .......... .......... ..........
GB-KOR2        .......... .......... .......... .......... .......... .......... .......... .......... .......... ..........
GN-KOR2        .......... .......... .......... .......... .......... .......... .....G.... .......... .......... ..........
JB-KOR2        .......... .......... .......... .......... .......... .......... .......... .......... .......... ..........
JB-KOR1        .......... .......... .......... .......... .......... .......... .......... .......... .......... ..........
GW-KOR1        .......... .......... .......... .......... .......... .......... .......... .......... .......... ..........
CN-KOR2        .......... .......... .......... .......... .......... .......... .......... .......... .......... ..........
CN-KOR3        .......... .......... .......... .......... .......... .......... .......... .......... .......... ..........
JB-KOR3        .......... .......... .......... .......... .......... .......... .......... .......... .......... ..........

              ....|....| ....|....| ....|....| ....|....| ....|....| ....|....| ....|....| ....|....| ....|....| ....|....|
                    910        920        930        940        950        960        970        980        990       1000
MN073839.1-Ch  ATGTTAACTT CACCCCTTCT TTTTATTGAT CTTTAGGTTT TGTTTTTTTA TTTACTGTGG GGGGTTTAAC TGGTGTTATT TTATCTAATT CTTCATTGGA
NC048990.1-Ch  .......... .......... .......... .......... .......... .......... .......... .......... .......... ..........
KY922482.1-UK  .......... T......... .......... .......... .......... .......... .......... .......... .......... ..........
GN-KOR1        .......... .......... .......... .......... .......... .......... .......... .......... .......... ..........
JN-KOR2        .......... .......... .......... .......... .......... .......... .......... .......... .......... ..........
GN-KOR3        .......... .......... .......... .......... .......... .......... .......... .......... .......... ..........
GB-KOR1        .......... .......... .......... .......... .......... .......... .......... .......... .......... ..........
JN-KOR4        .......... .......... .......... .......... .......... .......... .......... .......... .......... ..........
JN-KOR1        .......... .......... .......... .......... .......... .......... .......... .......... .......... ..........
GG-KOR1        .......... T......... .......... .......... .......... .......... .......... .......... .....C.... ..........
CN-KOR1        .......... .......... .......... .......... .......... .......... .......... .......... .......... ..........
CB-KOR1        .......... .......... .......... .......... .......... .......... .......... .......... .......... ..........
CB-KOR2        .......... .......... .......... .......... .......... .......... .......... .......... .......... ..........
JN-KOR3        .......... .......... .......... .......... .......... .......... .......... .......... .......... ..........
GB-KOR2        .......... .......... .......... .......... .......... .......... .......... .......... .......... ..........
GN-KOR2        .......... .......... .......... .......... .......... .......... .......... .......... .......... ..........
JB-KOR2        .......... .......... .......... .......... .......... .......... .......... .......... .......... ..........
JB-KOR1        .......... .......... .......... .......... .......A- -......... .......... .......... .......... ..........
GW-KOR1        .......... .......... .......... .......... .......... .......... .......... .......... .......... ..........
CN-KOR2        .......... .......... .......... .......... .......... .......... .......... .......... .......... ..........
CN-KOR3        .......... .......... .......... .......... .......... .......... .......... .......... .......... ..........
JB-KOR3        .......... .......... .......... .......... .......... .......... .......... .......... .......... ..........

              ....|....| ....|....| ....|....| ....|....| ....|....| ....|....| ....|....| ....|....| ....|....| ....|....|
                   1010       1020       1030       1040       1050       1060       1070       1080       1090       1100
MN073839.1-Ch  TGTTTCTATA CACGATACAT ACTATGTTGT AGCTCATTTC CATTATGTTT TATCTATGGG GGCTGTTTTT GCTATTATAT CAGGTATTAC CCATTGATTC
NC048990.1-Ch  .......... .......... .......... .......... .......... .......... .......... .......... .......... ..........
KY922482.1-UK  .......... .......... .......... .......... .......... .......... .....C.... .......... .......... ..........
GN-KOR1        ....C..... .......... .......... .......... .......... .......... .....C.... .......... .......... ..........
JN-KOR2        .......... .......... .......... .......... .......... .......... .....C.... .......... .......... ..........
GN-KOR3        .......... .......... .......... .......... .C........ .......... .....C.... .......... .......... ..........
GB-KOR1        .......... .......... .......... .......... .C........ .......... .....C.... .......... .......... ..........
JN-KOR4        .......... .......... .......... .......... .......... .......... .....C.... .......... .......... ..........
JN-KOR1        .......... .......... .......... .......... .......... .......... .....C.... .......... .......... ..........
GG-KOR1        .......... .......... .......... .......... .C........ .......... .....C.... .......... .......... ..........
CN-KOR1        .......... .......... .......... .......... .......... .......... .....C.... .......... .......... ..........
CB-KOR1        .......... .......... .......... .......... .......... .......... .....C.... .......... .......... ..........
CB-KOR2        .......... .......... .......... .......... .......... .......... .....C.... .......... .......... ..........
JN-KOR3        .......... .......... .......... .......... .......... .......... .....C.... .......... .......... ..........
GB-KOR2        .......... .......... .......... .......... .C........ .......... .....C.... .......... .......... ..........
GN-KOR2        .......... .......... .......... .......... .......... .......... .....C.... .......... .......... ..........
JB-KOR2        .......... .....C.... .......... .......... .C........ .......... .....C.... .......... .......... ..........
JB-KOR1        .......... .......... .......... .......... .C........ .......... .....C.... .......... .......... ..........
GW-KOR1        .......... .......... .......... .......... .......... .......... .....C.... .......... .......... ..........
CN-KOR2        .......... .....C.... .......... .......... .C........ .......... .....C.... .......... .......... ..........
CN-KOR3        .......... .....C.... .......... .......... .C........ .......... .....C.... .......... .......... ..........
JB-KOR3        .......... .......... .......... .......... .C........ .......... .....C.... .......... .......... .........T

              ....|....| ....|....| ....|....| ....|..
                   1110       1120       1130
MN073839.1-Ch  CCTTACTCTT ATAATATGCC TATAAACCCT TACTATT
NC048990.1-Ch  .......... .......... .......... .......
KY922482.1-UK  .......... .......... .......... .......
GN-KOR1        .......... .......... .......... .......
JN-KOR2        .......... .......... .......... .......
GN-KOR3        .......... .......... .......... .......
GB-KOR1        .......... .......... .......... .......
JN-KOR4        .......... .......... .......... .......
JN-KOR1        .......... .......... .......... .......
GG-KOR1        .......... .......... .......... .......
CN-KOR1        .......... .......... .......... .......
CB-KOR1        .......... .......... .......... .......
CB-KOR2        .......... .......... .......... .......
JN-KOR3        .......... .......... .......... .......
GB-KOR2        .......... .......... .......... .......
GN-KOR2        .......... .......... .......... .......
JB-KOR2        .......... .......A.. .......... .......
JB-KOR1        .......... .......... .......... .......
GW-KOR1        .......... .......... .......... .......
CN-KOR2        .......... .......... .......... .......
CN-KOR3        .......... .......... .......... .......
JB-KOR3        .......... .......A.. .......... .......
```

Figure S1. Multiple alignments of the differing nucleotide positions in the *COI* gene of *Carpoglyphus lactis*. A comparison was conducted between the *COI* sequence (1,137 nucleotides) of *C. lactis* mite isolated from the ROK in this study and the published sequence in GenBank. The highlighted blue frame signified shared nucleotide differences between the isolated strains and those from China (NCBI accession nos.: NC_048990.1, MN073839.1) and the UK (NCBI accession no. KY922482.1).
